# Angular Approach Scanning Ion Conductance Microscopy

**DOI:** 10.1016/j.bpj.2016.04.017

**Published:** 2016-05-24

**Authors:** Andrew Shevchuk, Sergiy Tokar, Sahana Gopal, Jose L. Sanchez-Alonso, Andrei I. Tarasov, A. Catalina Vélez-Ortega, Ciro Chiappini, Patrik Rorsman, Molly M. Stevens, Julia Gorelik, Gregory I. Frolenkov, David Klenerman, Yuri E. Korchev

**Affiliations:** 1 Department of Medicine, Imperial College London, London, United Kingdom; 2 Rayne Institute, King’s College London, London, United Kingdom; 3 Department of Materials and Department of Bioengineering and Institute for Biomedical Engineering, Imperial College London, London, United Kingdom; 4 National Heart and Lung Institute and Department of Cardiac Medicine, Imperial Center for Translational and Experimental Medicine, Imperial College London, London, United Kingdom; 5 Oxford Centre for Diabetes, Endocrinology, and Metabolism, University of Oxford, Churchill Hospital, Oxford, United Kingdom; 6 Department of Physiology, University of Kentucky, Lexington, Kentucky; 7 Department of Chemistry, University of Cambridge, Cambridge, United Kingdom

## Abstract

Scanning ion conductance microscopy (SICM) is a super-resolution live imaging technique that uses a glass nanopipette as an imaging probe to produce three-dimensional (3D) images of cell surface. SICM can be used to analyze cell morphology at nanoscale, follow membrane dynamics, precisely position an imaging nanopipette close to a structure of interest, and use it to obtain ion channel recordings or locally apply stimuli or drugs. Practical implementations of these SICM advantages, however, are often complicated due to the limitations of currently available SICM systems that inherited their design from other scanning probe microscopes in which the scan assembly is placed right above the specimen. Such arrangement makes the setting of optimal illumination necessary for phase contrast or the use of high magnification upright optics difficult. Here, we describe the designs that allow mounting SICM scan head on a standard patch-clamp micromanipulator and imaging the sample at an adjustable approach angle. This angle could be as shallow as the approach angle of a patch-clamp pipette between a water immersion objective and the specimen. Using this angular approach SICM, we obtained topographical images of cells grown on nontransparent nanoneedle arrays, of islets of Langerhans, and of hippocampal neurons under upright optical microscope. We also imaged previously inaccessible areas of cells such as the side surfaces of the hair cell stereocilia and the intercalated disks of isolated cardiac myocytes, and performed targeted patch-clamp recordings from the latter. Thus, our new, to our knowledge, angular approach SICM allows imaging of living cells on nontransparent substrates and a seamless integration with most patch-clamp setups on either inverted or upright microscopes, which would facilitate research in cell biophysics and physiology.

## Introduction

Monitoring molecular phenomena at precisely defined cellular locations, as opposed to the entire cell or the bulk of tissue, has become essential for understanding cellular mechanisms. However, imaging or manipulating live biological cells with nanometer resolution is a challenging task. It becomes more so when cells form tissues and organs that lack transparency and contrast, or are grown on nontransparent, often highly reflective substrates such as chips and micropatterns. Several super-resolution optical approaches have been developed to resolve structures beyond the diffraction limit, including single-molecule tracing (1), photoactivation localization microscopy (2), stochastic optical reconstruction microscopy (3), structured illumination microscopy (4), stimulated emission depletion microscopy (5), or pulsed two-photon stimulated emission depletion microscopy that allows high resolution imaging deep into the tissues (6). However, implementations of these techniques for imaging of living cells are still limited, also because of light-induced cell damage (7, 8). In addition, they all require fluorescent tags attached to the proteins or lipids, therefore showing nothing but structures of interest. In fluorescence-guided micromanipulations, this often causes probe misguidance resulting, for example, in patch-clamp recordings being made from invisible nonlabeled structures that cover fluorescently labeled active synaptic boutons rather than the presynaptic membrane itself (9). In contrast, confocal reflection microscopy provides information from unstained tissues. A combination of fluorescence confocal microscopy (FCM) and confocal reflection microscopy was successfully used to produce images of immunofluorescently labeled glial cells grown on a silicon substratum that is patterned with small 1-*μ*m-high pillars (10). Although the majority of fluorescence microscopy modalities use a configuration where the excitation of the fluorophore and detection of the fluorescence are done through the same light path/objective, its application to imaging of fluorescently labeled cells on nontransparent substrates using upright microscopy remains limited due to the high background produced by the excitation light reflected from the substrate.

In addition to nanoscale imaging, cell physiology experiments require nanoscale manipulations. A micropipette has been widely used for local application of chemicals, rapid perfusion application, intra- and extracellular perfusions, voltage measurements, whole-cell and single-channel current recording, etc. However, until now researchers have routinely relied on the far-field microscopy to navigate the micropipette probe. This provided positioning accuracy of only or just under a micrometer, even if sharp micropipettes (tip diameter 20–50 nm) were used to study local nanoscale phenomena. In the meantime, we have been developing an alternative pipette positioning-and-probing technique—scanning ion conductance microscopy (SICM) (11, 12, 13, 14). Changes in pipette current provide a feedback signal that allows a nanopipette to hover over a biological sample. This technique can generate topographic images of live cells with a resolution of ∼10 nm, the highest achieved in live preparations (15). In contrast to other methodologies, SICM combines nanoscale imaging of live cellular structures with the power of functional assays by nanopipette tools.

The combination of nanoscale imaging, recording, and manipulation that SICM can deliver represents a paradigm shift, allowing experimenters to record efficiently from cellular micro- and nanostructures such as synapses, t-tubules, microvilli, or fine dendrites that are simply too small for navigation with conventional optical techniques. The SICM probe is ideally suited for performing nanoscale assays on the cell surface, that include patch-clamp recordings from individual surface structures (16, 17, 18, 19, 20), ionophoretic delivery of reagents (21), or pressure microapplication via the pipette to explore mechanical properties of the cell or to deliver reagents (22, 23).

Hopping probe ion conductance microscopy (HPICM) is a version of SICM that was developed specifically for imaging of various specialized differentiated cells and tissues (13). These cells often have very convoluted surfaces with vertical dimensions up to a hundred micrometers. HPICM uses an electrolyte-filled glass nanopipette with an inner Ag/AgCl electrode as an imaging probe. The pipette is mounted with its tapered end facing downward on a linear piezo actuator that provides vertical movement (Z piezo). A sample, such as cells or tissue, is typically located at the bottom of a petri dish in physiological solution. The system continuously monitors the ion current that flows between the measuring electrode (nanopipette) and the reference electrode in the dish. This current drops when the pipette approaches the sample surface from above to a distance shorter than the radius of the pipette opening. At this time, the Z piezo position is stored as a pixel of topographical image representing the sample height at this particular location. The pipette is then withdrawn and the XY piezo stage moves the sample to a new measurement point. Because the drop of the ion current is detected at a certain distance from the surface, the HPICM ensures no direct physical contact between the nanopipette and the sample. The ability to visualize very convoluted structures in physiological conditions makes HPICM a universal tool for nanoscale surface imaging of various cell types previously inaccessible to scanning probe techniques (13).

Despite remarkable success in imaging previously inaccessible structures, HPICM inherited a common design of SICM setups based on the vertical alignment of the nanopipette probe. This design assumes mounting of the piezo assembly on an inverted microscope, right above the objective, which results in significant compromises in the optical imaging by prohibiting high numerical aperture condensers, phase contrast, or differential interference contrast (DIC). Obviously, this design is also incompatible with the upright microscopes that are widely used for studies of cell function in tissue slices or for in vivo imaging. Therefore, all currently available SICM implementations represent dedicated setups with or without additional functions, but not an integration of the SICM capabilities into the existing cell physiology rigs. The goal of this proof of principle study was to design a HPICM that would allow imaging of the samples at an arbitrary angle and its seamless integration with existing patch clamp and/or imaging systems.

## Materials and Methods

### Measurements of the probe current

MultiClamp 700B or Axopatch 200B amplifiers were used for ion current measurements (Molecular Devices, Wokingham, UK). Typically, a bias potential of 200 mV was used for imaging. Traces were analyzed using pClamp 10 (Molecular Devices). Pipettes with estimated inner diameters of 50–100 nm were pulled from borosilicate glass (O.D. 1 mm, I.D. 0.5, Intracel, Cambridge, UK or World Precision Instruments, Hitchin, UK) using a P-2000 laser puller (Sutter Instruments, Novato, CA). For imaging experiments, the nanopipettes were filled with the corresponding bath solutions.

### Nanoneedle arrays

Porous silicon nanoneedles were fabricated on 100 mm diameter, 0.01 Ω-cm, p-type silicon wafers by microfabrication as previously described (24, 25). First, a 160 nm layer of silicon-rich silicon nitride was deposited by low-pressure chemical vapor deposition on the substrate, followed by spin coating of 250 nm thick negative-tone photoresist (NR9-250P, Futurrex, Franklin, NJ). The photoresist was patterned with an array of 600 nm diameter dots with 2 *μ*m pitch by means of 1X ultraviolet photolithography in an MA-6 mask aligner (Karl Suss, Germany). The pattern was transferred into the silicon nitride layer by reactive ion etch for 120 s in an PlasmaPro NPG 80 (Oxford Instruments, Yatton, UK), 20 sccm CF_4_, 5 sccm O_2,_ 100 mTorr, 200 W RF. Following pattern transfer, the photoresist was stripped in acetone, followed by cleaning with methanol and isopropanol, and the remaining organic residues were removed with 10 min of O_2_ plasma in a barrel asher (Diener plasma technologies, Ebhausen, Germany). The native oxide layer over the Si was removed by a 1 min dip in 10% v/v HF. Silver nanoparticles were selectively deposited on the exposed Si by electroless deposition from an aqueous solution of 0.02 M AgNO_3_ and 10% v/v HF. The substrate was rinsed with excess water, washed in isopropanol, and dried under N_2_ stream. Porous silicon pillars with 600 nm diameter were formed by metal-assisted chemical etching of the substrate in 400 ml aqueous solution with 80 ml of HF and 5 ml of H_2_O_2_ for 7 min. The substrate was washed with excess water, rinsed with isopropanol, and dried under N_2_ stream. The pillars were shaped into the conical nanoneedles by reactive ion etching for 2 min and 30 s in an PlasmaPro NPG 80, 20 sccm SF_5_, 100 mTorr, 240W RF. The substrate was then diced into 8 × 8 mm chips in a dicing saw and the chips were oxidized by O_2_ plasma for 10 min in a barrel asher (Diener plasma technologies). The oxidized substrates were functionalized with 2% v/v 3-aminopropyltriethoxysilane in ethanol for 2 h, rinsed thrice in ethanol, and dried under N_2_ stream.

### Culture and preparation of human mesenchymal stem cells, human umbilical vein endothelial cells, and HeLa cells

Human mesenchymal stem cells (hMSCs) were purchased from a commercial source (Lonza Biologics, Slough, UK) and expanded up to passage number 3. These cells were derived from human bone marrow of a Caucasian female aged 29. hMSCs were cultured in Alpha Minimum Essential medium (Invitrogen, Carlsbad, CA) supplemented with 20% fetal bovine serum (FBS) and 1% Antibiotic-Antimycotic (Thermo Fisher Scientific, East Grinstead, UK). Human umbilical vein endothelial cells (HUVECs) were cultured in endothelial basal medium supplemented with EGM2 SingleQuot Kit supplement and growth factors. HeLa cells were cultured in Dulbecco’s modified essential medium (Thermo Fisher Scientific) supplemented with 10% fetal calf serum and 1% Antibiotic-Antimycotic. Cells were cultured at 37°C and 95% air/ 5% CO_2_ with the medium being changed every other day.

For fixed cell experiments, media was aspirated and samples were washed with phosphate buffered saline (PBS, Invitrogen) before fixing with 3.7% paraformaldehyde solution after 6 h of culture on nanoneedle arrays (NNAs). For visualization under an upright microscope, cells were incubated with 0.5*μ*g/ml of wheat germ agglutinin (WGA) Alexafluor 488 Conjugate (Thermo Fisher Scientific) for 10 min and washed in PBS. For live experiments, cells were stained with WGA and then supplemented with Lebovitz-15 media at 37°C (Thermo Fisher Scientific).

### Islets of Langerhans

Pancreatic islets were isolated from NMRI mice by injecting collagenase solution into the bile duct, with subsequent digestion of the connective and exocrine pancreatic tissue. Islets were picked using a P20 pipette, under a dissection microscope. Native islet preparations were cultured at 37°C, 5% CO_2_, absolute humidity in RPMI-1640 medium supplemented with 11 mM glucose, 10% FBS, 100 units/mL of penicillin and 100 *μ*g/mL of streptomycin. Animal surgical procedures and perioperative management conformed to the UK Animals (Scientific Procedures) Act 1986.

### Isolated adult rat cardiac myocytes

Adult male Sprague-Dawley rats (250–300 g) cardiomyocytes were isolated by the Langendorff perfusion method, as previously described (26). After isolation, cardiomyocytes from the left ventricle were plated on laminin-coated coverslips and left to adhere for at least 45 min before the start of experiments. Cardiomyocytes were used on the same day of isolation. Cells were washed once with the external recording solution and mounted on the microscope stage for electrophysiological recordings. Animal surgical procedures and perioperative management conformed to the UK Animals (Scientific Procedures) Act 1986. Imperial College London Ethical Review Committee and the project license authorized these studies in accordance with the United Kingdom Home Office Animals (Scientific Procedures) Act 1986.

### Hippocampal neurons preparation

Rat hippocampal neurons were kindly provided by Dr. K. Volynski, Institute of Neurology, UCL and prepared as previously described (27) and cultured on glass coverslips. Cells were kept in an incubator at 37°C and 95% air/5% CO_2_ for 1–2 weeks. Once out of the incubator, cells were washed with standard external solution and imaged within 2 h, at room temperature. Animal procedures were done in compliance with Home Office (UK) regulations and Animals (Scientific Procedures) Act, 1986 and approved by the local animal ethics committee of University College London.

### Cultured organs of Corti

Organ of Corti explants were dissected from wild-type C57BL6 mice at postnatal day 3 (P3) and placed in glass bottom petri dishes (WillCo Wells, Amsterdam, The Netherlands). The explants were cultured in Dulbecco’s modified essential medium supplemented with 25 mM HEPES, 7% FBS (Invitrogen) and 10 mg/L of ampicillin (Calbiochem, La Jolla, CA) at 37°C and 95% air/5% CO_2_. After 2–3 days in culture, the organs of Corti explants were either imaged live or fixed in 2.5% glutaraldehyde in 0.1 M cacodylate buffer supplemented with 2 mM CaCl_2_ for 1–2 h at room temperature. During imaging, the fixed explants were kept in Hank’s balanced salt solution (Thermo Fisher Scientific) and live explants in L-15 medium (Thermo Fisher Scientific). Animal procedures were approved by the University of Kentucky Animal Care and Use Committee.

### Cell-attached electrophysiological recordings

Cell-attached patch-clamp recordings of K_ATP_ channels were performed at room temperature using the following solutions; external solution containing: 145 mmol/L KCl, 1 mmol/L MgCl_2_, 1 mmol/L CaCl_2_, 2 mmol/L EGTA, 10 mmol/L Glucose, 10 mmol/L HEPES, pH 7.4 with KOH; internal recording solution containing in: 145 mmol/L KCl, 2 mmol/L MgCl_2_, 5 mmol/L HEPES, pH 7.4 with KOH. The pipette used for cell attached recordings had a typical resistance of ∼40 MΩ. Currents were recorded using Axopatch 200A amplifier (Axon Instruments, Foster City, CA), controlled and monitored using pClamp software version 10 (Axon Instruments). To generate a current-voltage (I-V) relationship, the membrane under the patch was held at a voltage of 0 mV and incremental positive or negative steps of 20 mV were applied to 100 and −100 mV. Data were low-pass filtered at 1 kHz using the built-in Bessel filter of the amplifier and sampled at 20 kHz. A Liquid junction potential, calculated to be 0.2 mV, was not corrected from the data shown due to its negligible effect.

### Scanning electron microscopy

HeLa cells were fixed in 0.2% w/v glutaraldehyde in PBS at the required time point for 15 min and washed three times with PBS (see Fig. 2 *H*).

hMSCs were fixed in 0.25% w/v glutaraldehyde in 0.1 M sodium cacodylate buffer (Electron Microscopy Sciences, Hatfield, PA) at the required time point for 45 min and washed three times in sodium cacodylate buffer (see Fig. 2 *I*). Samples were then treated with 1% Osmium tetroxide (Electron Microscopy Sciences) in sodium cacodylate buffer for 1 h followed by two washes in double-distilled water for 10 min. Samples were treated with 1% (w/v) tannic acid in water and subsequently washed in distilled water twice for 10 min.

Samples were then dehydrated in a series of graded ethanol (10%, 30%, 50%, 70%, 90%, 2 × 100%, v/v). Samples were treated with hexamethyldisilazane (Sigma-Aldrich, Gillingham, UK) for 5 min and allowed to air dry. Samples were then mounted on stubs and sputter coated with 10 nm of chromium to improve conductivity, and analyzed using a LEO 1525 field emission-scanning electron microscope (SEM) gun.

## Results

**Figure 1:**
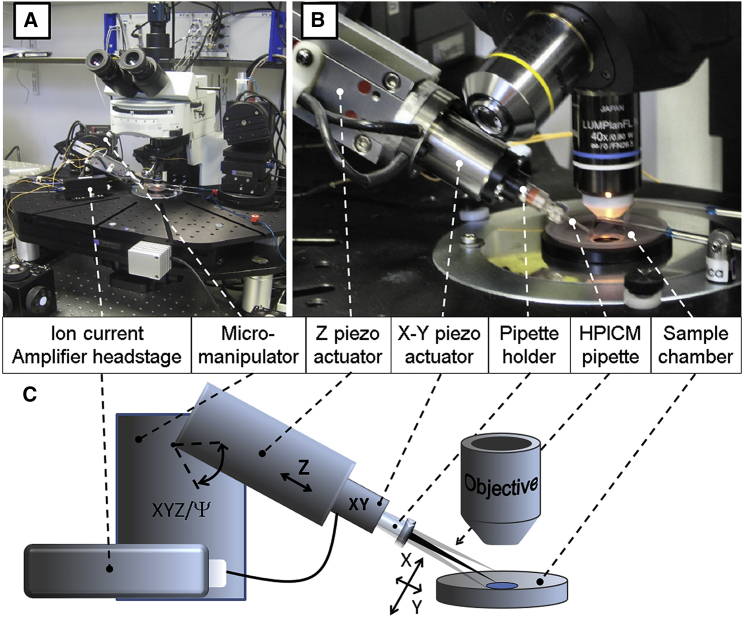
Photographs and schematic diagram of experimental setup. (*A*) A photograph of the HPICM rig mounted on an upright microscope. (*B*) Close up view of Z and XY piezo assembly and scanning nanopipette targeting cell sample. (*C*) Schematic diagram showing the HPICM key components and the movements in *X*, *Y*, and *Z* directions that could be achieved by tilting the nanopipette and moving it along the *Z* axis. To see this figure in color, go online.

We have built a HPICM with an adjustable nanopipette approach angle that can be integrated into any patch-clamp setup, including the setups with an upright optical microscope (Fig. 1). High-resolution HPICM scanning was performed by a three-dimensional (3D) piezo actuator assembly mounted on a PatchStar micromanipulator (Scientifica, Uckfield, UK). The micromanipulator provided a coarse approach and positioning for the HPICM nanopipette over a 20 mm range in *X*, *Y*, and *Z* directions that covered most of the 35 mm diameter petri dish sample area and also allowed the complete withdrawal of the nanopipette, which is necessary for sample change.

The approach angle of the micromanipulator can be adjusted between 0° and 90° to the horizontal plane, which allows imaging under high magnification objectives that have a short working distance. The approach angle of 33° to the surface was experimentally selected as the optimal angle for scanning under the Olympus water immersion objective LUMPlanFL N 40×, 0.8 numerical aperture, 3.3 mm working distance. The PatchStar micromanipulator and the sample holder were mounted on the Motorized Movable Top Plate (Scientifica, UK) that provided the coarse positioning required for the selection of the area of interest. In our HPICM for upright optical microscopes we implemented a pipette-scanning design where the entire XYZ scanning was performed by the nanopipette while the sample remained in a fixed position. Such design makes the integration of HPICM with standard patch clamp rigs found in thousands of laboratories around the world straightforward. It also enables one or more extra pipettes to be installed for either whole-cell recordings or localized delivery as previously demonstrated for inverted optical microscopes (20). The XYZ piezo assembly consisted of an S-316.10 tip-tilt piezo scanner (Physik Instrumente, Karlsruhe, Germany) that provided scanning in the *XY*-plane and was mounted on the end face of a P-753.3CD piezo actuator (Physik Instrumente) that delivered 38 *μ*m travels in the *Z* axis. At a full 1200 *μ*rad tilt angle of S-316.10 and a pipette assembly of 50 mm length (when measured from the piezo platform plane to the scanning pipette tip), a 40 × 40 *μ*m size scan in the *XY*-plane was achieved. Both, P-753.3CD *Z* axis and S-316.10 *XY* axes piezos were tuned to a 10 milliseconds time response to ensure rapid, resonance-free imaging. An adaptor coupler was designed to mount a standard EPS series microelectrode holder (Warner Instruments, Hamden, CT) onto the S-316.10 piezo moving platform. A microelectrode holder model ESP-F10P with the side pressure port (Harvard Apparatus, Cambridge, UK) was used to enable patch-clamp recordings.

An alternative HPICM, also with an adjustable nanopipette approach angle, was built independently at the University of Kentucky for integration into a standard patch-clamp setup on an inverted microscope. In this HPICM, the hopping (known as “Z” in the classical design) movement was provided by a ring piezoactuator (RA 12/24 SG) driven by two ENV 800 SG amplifiers working in parallel (Piezosystem, Jena, Germany), whereas movement in the plane perpendicular to the hopping (“XY” in the classical design) was provided by the TRITOR 38 SG translation stage driven by EVD 125 digital amplifiers (Piezosystem). The XYZ piezo assembly was mounted on a compact rotary stage (#55-030, Edmund Optics, Barrington, NJ), which in turn was mounted on a standard patch-clamp micromanipulator (MP-285, Sutter Instruments).

Both angular approach HPICMs produced similar imaging results on a variety of samples without interfering with optical imaging of the specimens. The only difference was in the software modification that was required to recalculate the X-Y commands into the combination of tilt angles and Z-corrections to properly drive the S-316.10 tilt scanner (see Fig. 1 *C*). Obviously, this modification was not needed for the linear TRITOR 38 SG translation stage of the other HPICM setup (not shown).

To demonstrate the applicability and robustness of the angular approach HPICM for imaging of cells grown on nontransparent substrates and small organs we scanned HUVECs and hMSCs seeded on NNAs, as well as islets of Langerhans, cultured organs of Corti, primary hippocampal neurons, and intercalated disks of isolated cardiac myocytes. The angular HPICM imaging required no specific sample preparation. A person with previous experience in SICM could spend no more than 5 min from mounting the sample onto the stage to start scanning.

### HPICM imaging of samples on nontransparent substrates

Here, we demonstrate the applicability of the HPICM to image cells cultured on novel, to our knowledge, biocompatible substrates that are nontransparent. In the last decade, high aspect ratio nanowire or NNAs have emerged as a promising platform for delivery of genetic material and cell-impermeable biomolecules, as well as intracellular sensing by increasing the physical accessibility to the cell interior (24, 28, 29, 30, 31, 32, 33). Because of its large internal surface area and biocompatibility, porous silicon has recently been used to create vertical NNAs (50 nm diameter tip, 600 nm base, 4 *μ*m height). Porous silicon NNA possess a high loading capacity and are amenable to accommodate a variety of biomolecules and nanoparticles (24, 25, 28, 34).

Although NNA can probe the cell interior without compromising their viability, cells show unique behaviors and morphologies on NNA. For example, cells show reduced migration and spreading along with stronger adhesion to NNAs in comparison to flat surfaces (35). Although increased adhesion is seen to improve cytosolic cross talk at the cell-nanoneedle interface, the specific processes that lead to delivery and intracellular sensing remain controversial (32, 33, 36, 37). Although it has been suggested that the efficient delivery of a range of biological (32) or cell-impermeable (38) agents occurs via physical penetration of the membrane, conflicting evidence suggests that NNA are engulfed by cells and activation of endolysosomal pathways may be facilitated. Penetration of high aspect ratio nanostructures through plasma membrane has been recently demonstrated by building a transparent substrate consisting of a nanostraw array integrated with a microfluidic device, so that live imaging could be performed using an inverted epifluorescence microscope (38). The majority of NNAs, however, are produced on nontransparent substrates making it very difficult to perform live cell imaging (24, 31, 39). Current studies of NNA-cell interface are limited as they have largely relied on fixed cells limiting the investigation of the dynamic, real-time processes of membrane permeability, cell attachment and spreading, and uptake mechanisms. Furthermore, it is well documented that dehydration and fixation protocols required for SEM imaging cause cell shrinkage and detachment from the substrate introducing numerous artifacts (40). Additionally, noncontact, subnanometer, label-free live studies of cells in their native environment on NNA are yet to be presented.

**Figure 2:**
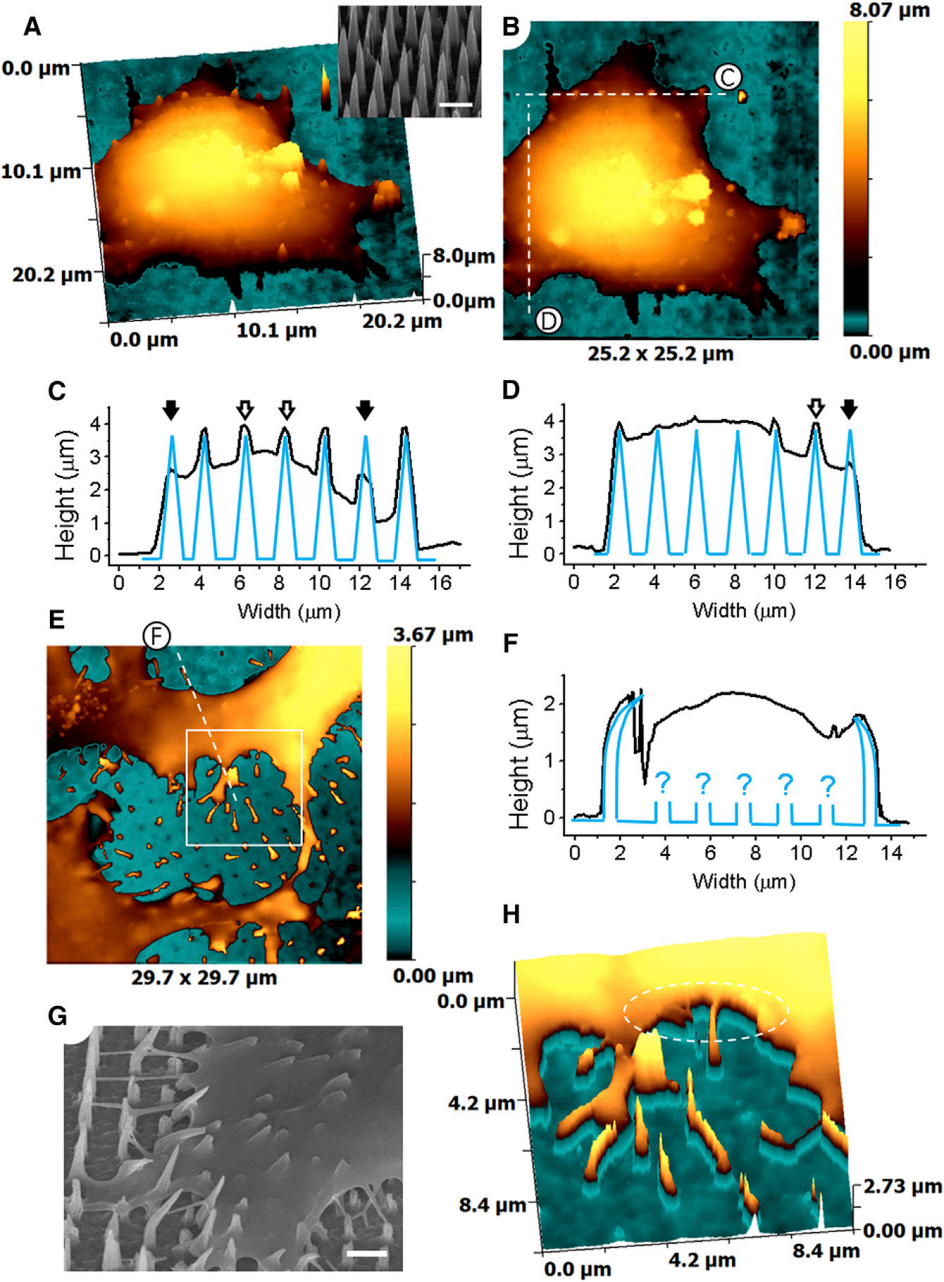
Images of HUVEC cells grown on NNA acquired using standard HPICM with vertical approach. (*A*) 3D representation of a HUVEC cell grown on NNA for 4 h. Nanoneedles surrounding the cell are not resolved due to their low aspect ratio that results in steep slopes. Inset shows SEM image of NNA. The scale bar represents 2 *μ*m. (*B*). 2D representation of image shown in (*A*). (*C* and *D*) Cross-section profiles plotted at corresponding dashed lines in (*B*) (*black trace*) and schematic representation of nanoneedles (*blue*) showing full height NNAs wrapped in the cell membrane (*hollow arrows*) and NNAs, which pierce the cell (*solid arrows*). (*E*) HUVEC cell grown on NNA for 48 h. (*F*) Cross-section profiles plotted at dashed line in *E* (*black trace*) and schematic representation of nanoneedles (*blue*). (*G*) SEM image of a HeLa cell grown on NNA for 8 h. The scale bar represents 2 *μ*m. (*H*) High-resolution scan of the area boxed in (*E*) showing bent nanoneedles. To see this figure in color, go online.

We first used our standard (vertical approach) HPICM built around an inverted microscope to image live cells grown on NNAs. Lacking the optical guidance due to the opacity of the NNA we blindly approached the sample and performed imaging at random positions until we located a cell. An HPICM image (representative of three images, *n* = 3) of a fixed HUVEC cell after being grown on NNA for 4 h is shown in 3D and two-dimensional (2D) projections in Fig. 2, *A* and *B*, correspondingly.

Based on the previously reported SEM data showing negligible degradation of pSi nanoneedles incubated in culture conditions for up to 8 h (24), we expect little or no NNA degradation to be seen in our HPICM images at 4 h time point. However, at the acquisition resolution of 200 nm per pixel, nanoneedles surrounding the cell are not visible due to their low aspect ratio 0.6/4.0 yielding steep slopes (4.3° angle to normal) (Fig. 2 *A*, *inset*). This is because the ion current reduction occurring when a vertically aligned pipette approaches slopes close to normal is below the detection limit of our microscope. Hence, the feedback control continues the approach until the pipette reaches the bottom of the array and the ion current drops below the specified set point. The ion current reduction during approach to different slopes has been previously described using finite element modeling by Del Linz et al. (41). Scanning over the tips of nanoneedles interfacing with cells revealed 4 *μ*m high peaks (Fig. 2, *C* and *D*, *hollow arrows*), which is the same as the intact nanoneedles height (Fig. 2, *A*, *inset*), as well as lower height, blunt protrusions (*solid arrows*). The 4 *μ*m high peaks most likely represent the apical cell membrane pinned above the tips of underlying nanoneedles, which widens nanoneedles making them easier to resolve. Given that the HPICM cannot resolve naked nanoneedle tips due to their small size and high aspect ratio, the lower peaks could represent the places of interaction between the nanoneedles and the cell membrane at lower heights where nanoneedles pierce the cell and protrude above. Alternatively, the lower protrusions may represent nanoneedles broken by the force applied to them by cells.

An HPICM image (representative of three images, *n* = 3) of a fixed HUVEC cell after being grown on NNA for 48 h acquired at two-level adaptive resolution of 110 and 220 nm per pixel (13) reveals either bent or broken nanoneedles (Fig. 2 *E*). The cross-section profile corresponding to the dashed line in Fig. 2 *E* most likely indicates the presence of bent nanoneedles on both edges of the cell (Fig. 2 *F*). It is impossible to say, however, what happened to the nanoneedles underneath the cell. For comparison, SEM images of HeLa cells spreading on nanoneedles after 8 h show strikingly similar behavior as they pull the NNA, bend and break the nanoneedles, especially closer to the cell periphery (Fig. 2 *G*). A high-resolution HPICM scan of the boxed area is shown in Fig. 2 *F*. The exact angle at which the edge of the cell is interacting with the NNA cannot be resolved due to the vertical approach of the scanning pipette and is seen as a vertical cliff (*dashed oval*).

**Figure 3:**
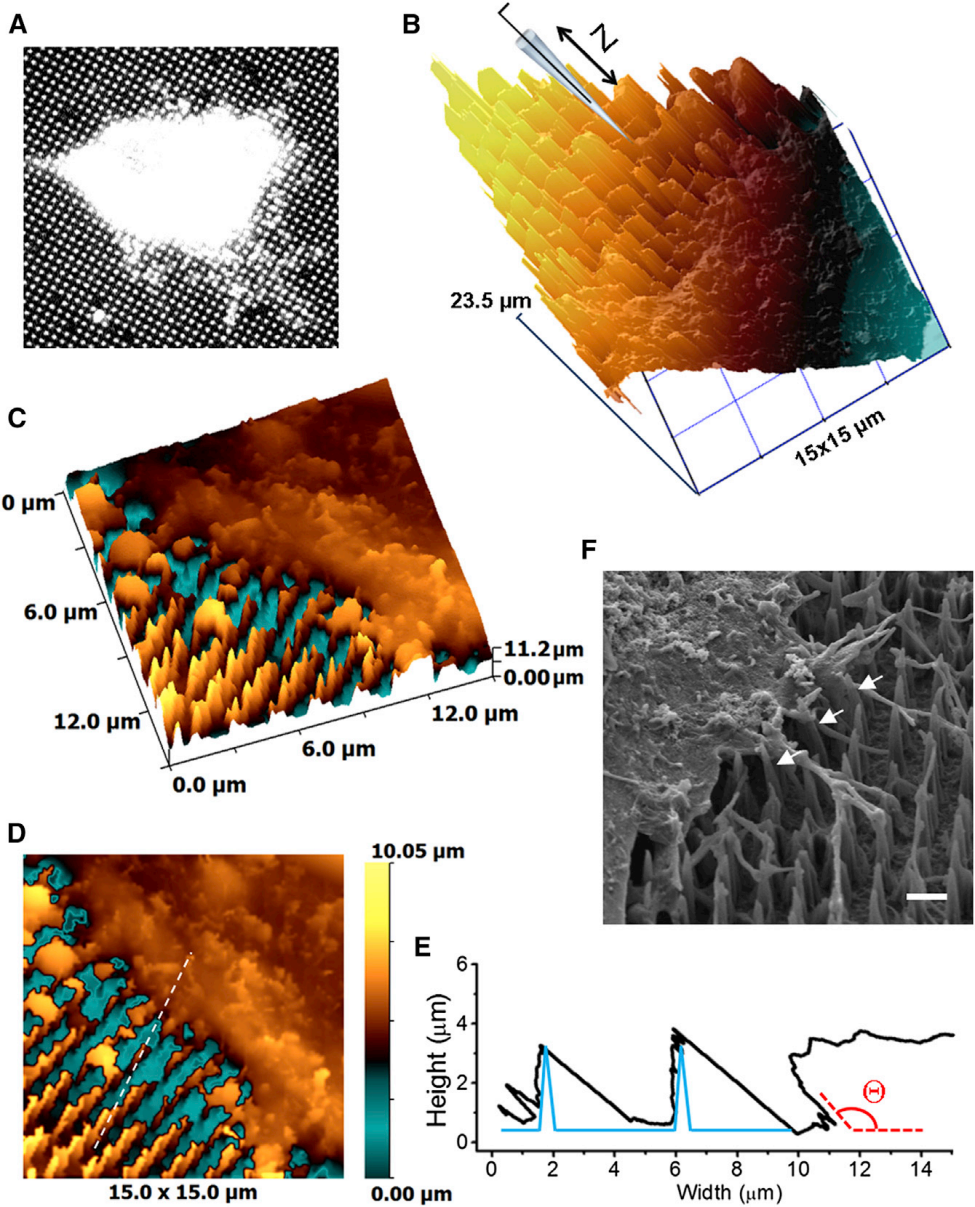
Images of live hMSCs growing on NNA acquired using HPICM with angle approach under upright microscope. (*A*) Fluorescent image of a live hMSC grown on NNA between 4 and 8 h, labeled with WGA and acquired using an upright microscope. (*B*) Montage of topographical image of hMSC grown on NNA and schematic representation of the HPICM pipette scanning under angle toward the array. (*C*) 3D representation of the slope corrected image shown in (*B*). (*D*) 2D representation of the slope corrected image shown in (*B*). (*E*) Cross-section profile corresponding to the dashed line shown in (*E*) plotted using slope-corrected data (*black trace*) and schematic representation of nanoneedles (*blue*) showing the angle at which the cell interacts with the substrate (Θ, *red*). (*F*) SEM image of an hMSC grown on NNA for 6 h prepared using osmium and tannic acid treatment showing bent NNAs (*white arrows*). The scale bar represents 2 *μ*m. To see this figure in color, go online.

In contrast to vertical approach HPICM that can only be used with inverted optics, HPICM constructed for an upright microscope enables identifying the location of cells grown on nontransparent substrates before imaging, for example by using fluorescent WGA staining (Fig. 3 *A*). A montage of a raw topographical image (*n* = 6) of a live hMSC growing on a NNA for 6 h and a schematic representation of the scanning pipette approaching at 33° to the surface is shown in Fig. 3 *B*. At such an approach angle nanoneedles are seen as steps. However, slope corrected and rotated to the observer (Fig. 3 *C*), the image gives nanoneedles visual appearance similar to their typical SEM projection (Fig. 2 *A*, *inset*). Cross-section profile (*black trace*) showing two nanoneedles and the cell edge corresponding to a dashed line in Fig. 3 *E* but plotted using raw, slope-corrected data is shown in Fig. 3 *F*. Note that although the detected angle Θ at which the cell interacts with the substrate is limited by the nanopipette approach angle, it is over 90° to substrate. For comparison, SEM image (*n* = 5) of an hMSC grown on NNA for 6 h is shown in Fig. 3 *F*. This time, to rule out the possibility of the fixation-induced cell shrinkage that may result in NNA bending, we prepared the samples of hMSCs using osmium and tannic acid treatment that stabilizes lipid membranes. The SEM images also reveal bent NNAs (*white arrows*). This is in agreement with the previously published observations showing that silicon nanowire arrays with Young’s modulus (*E*) = 151 GPa, the highest value of stiffness of a substrate that has so far been used to measure cell traction forces, could be bent by three different cell types exhibiting traction forces in the micronewton range (42). The traction forces exhibited by the cells are related to their migrational abilities, with cancerous cells being able to exert larger forces than mechanosensitive ones. Thus, we can predict that HeLa cells can produce a greater amount of traction force and pull on the NNA, compared to mechanosensitive cell types such as HUVECs and hMSCs. With respect to our porous silicon NNA, being a softer but more brittle material with *E* = 2.4 GPa, failure load (Pt) = 260 nN, and critical buckling load (Pcr) = 264 nN (Supplementary Fig. S2 in (24)), experiencing a traction force of cells in the micronewton range, we can predict that the bending and/or bucking of nanoneedles is likely to happen due to the cells themselves rather than an artifact of electron microscopy (EM) sample preparation as shown in the SEM images (Fig. 2 *H*). 3DEM analysis is also under investigation; however, it is outside the scope of this work.

Thus, we have demonstrated here that the angle scan HPICM was able to visualize extremely challenging areas of the cells grown on the nanoneedle substrate, which opens a possibility for their functional exploration using various previously developed SICM approaches (43). It is worth mentioning that imaging of such challenging samples has not been reported with any other type of scanning probe microscopy.

### HPICM imaging of islets of Langerhans

**Figure 4:**
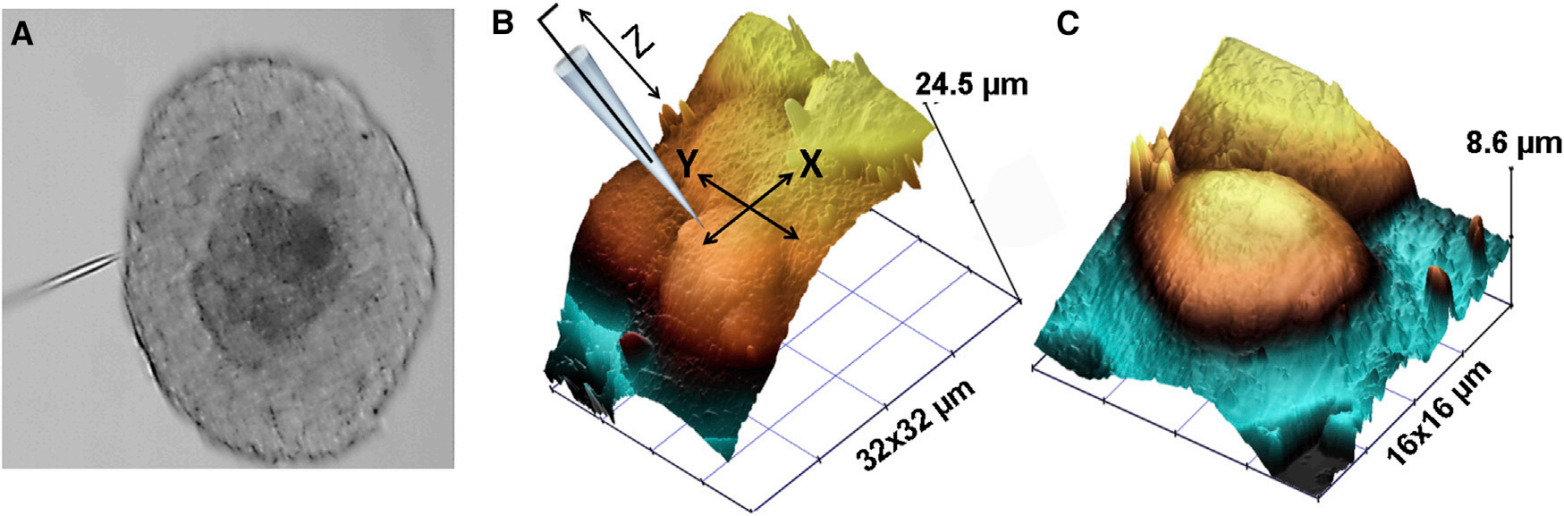
HPICM topographical imaging of islets of Langerhans. (*A*) Optical image of an islet and an HPICM scanning pipette acquired using a 60× water immersion objective. (*B*) Montage of an HPICM topographical image with a schematic representation of the scanning pipette. (*C*) 3D representation of adjacent *β*-cells in an islet. To see this figure in color, go online.

Here, we demonstrate that HPICM can image isolated islets of Langerhans under an upright microscope (Fig. 4). Isolated islets of Langerhans are the physiologically relevant model for studying insulin secretion. Insulin, a hormone that controls blood glucose levels, is implicated in diabetes mellitus, a disease characterized by elevated levels of blood glucose over a prolonged period. Diabetes can cause many life-threatening complications, both acutely and in long term (44). Insulin is produced by beta cells of pancreatic islets of Langerhans. Relatively little is known about the mechanisms and rates of single insulin granule secretion and dissolution. Comparing the release and dissolution rates and factors that affect them in health and disease will help to better understand basic physiology as well as causes of diabetes. However, the paucity of experimental methods capable of measuring insulin release and dissolution at the level of single vesicles complicates the research. Currently, available microscopy techniques either 1) enable very high resolution imaging of chemically fixed, mummified cells (such as EM) that does not allow to explore insulin granule release dynamics or 2) total internal reflection fluorescence microscopy that is indirect as it relies on the visualization of fluorescent tags, most of which exceed insulin in size thereby inevitably affecting its kinetics. Video rate two-photon FCM has been used to follow the release of insulin in islets of Langerhans (45, 46). However, diffraction limited lateral and 1 *μ*m axial resolution is barely sufficient to resolve details of individual insulin granule release using this imaging technique alone. Fig. 4 *A* shows optical image with HPICM pipette scanning islet of Langerhans (*fixed*).

Montage of raw HPICM topographical image (representative of 10, *n* = 10) and schematic representation of the scanning pipette is shown in Fig. 4 *B*. Individual *β*-cells comprising the islet can be seen as dome-shaped structures in the slope corrected image shown in Fig. 4 *C*. Mouse islets typically comprise *β*-cells (80%), *α*-cells (up to 17%), and *δ*-cells. *β*-cells can be distinguished from the other two types by their larger size and higher glucose activation threshold (>6 mM vs. 3 mM for *α*-cells). Previously, we have demonstrated that it is possible to follow the formation and internalization of individual clathrin-coated pits in living cells by correlative HPICM and FCM(47). The successful imaging with angled approach HPICM opens the possibility for a similar study directly on islets of Langerhans. More importantly, angled approach HPICM may allow studying insulin secretion in these cells at the single vesicle level using a combination of HPICM and recently reported video rate two-photon FLM(45, 46).

### HPICM imaging of vertical cellular projections

**Figure 5:**
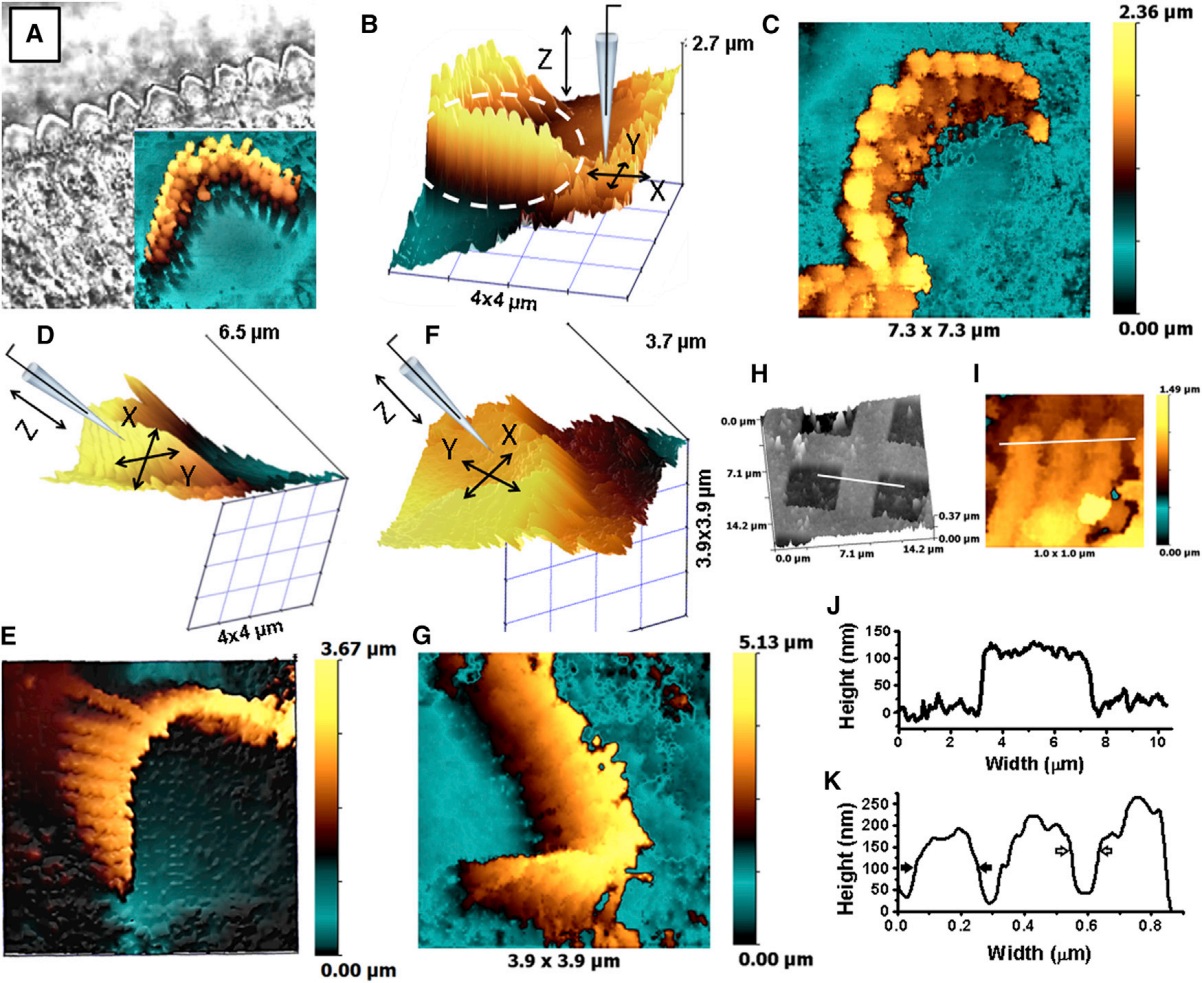
Imaging of the vertical stereocilia in the wild-type mouse auditory hair cells (*fixed*). (*A*) Optical image of an organ of Corti explant acquired with 60× water immersion objective showing a row of OHCs. Inset shows 2D projection of the topographical image acquired in (*B*). (*B*) Topographical image of an OHC stereocilia bundle acquired with a vertically mounted HPICM pipette. (*C*) 2D projection of the topographical image of a live IHC stereocilia bundle acquired with a vertically mounted HPICM pipette. (*D* and *F*) Montage of topographical images and the schematic representation of angular approach HPICM pipettes scanning OHC stereocilia bundle from the inhibitory (*D*) and excitatory (*F*) directions. Coordinate axes aligned with the axis of the pipette. (*E* and *G*) Projections of the topographical images presented in (*D*) and (*F*) showing vertical surfaces of stereocilia bundles from the inhibitory (*E*) and excitatory (*G*) directions. (*H*) An image of calibration sample with 100 nm vertical steps obtained with the same angular approach HPICM setup. (*I*) A high-resolution image of individual OHC stereocilia from the excitatory direction. (*J* and *K*) Cross-section profiles corresponding to (*H*) and (*J*), respectively. Solid and hollow arrows point at individual stereocilia width (200 nm) and gap between stereocilia (80 nm), respectively, measured as FWHM. To see this figure in color, go online.

To demonstrate the applicability of angular approach HPICM for imaging the structures that are inaccessible to other scanning probe techniques, we imaged the vertical surfaces of the mechanosensitive stereocilia of the inner ear hair cells that are arranged in rows of increasing height, forming a hair bundle (48). Conventional optical microscopy hardly resolves individual stereocilia, because their diameter could be about or <200 nm and, therefore, optical microscopy can often see only the overall V-shapes of the hair bundles at the surface of the organ of Corti explant (Fig. 5 *A*). Sound-induced deflections of these bundles open the mechanotransducer channels that are located somewhere at the tips of the shorter row stereocilia (49) and initiate the sequence of events that ultimately results in the sense of hearing. The molecular identity of mechanotransducer channels is currently a subject of intense debates, which are complicated by the fact that the auditory hair cells exhibit abnormal mechanotransduction in several pathological conditions (50, 51). Therefore, it is yet unclear whether these cells express several types of mechanosensitive channels or whether these channels could migrate along the apical surface of the hair cells (50, 52, 53). SICM could be very helpful in clarifying these questions, because it could localize and map the distribution of functional channels in a living cell (20). SICM has previously been used to provide guidance for targeted patch clamping with tens of nanometer resolution (9, 19, 54) and to make the first preliminary patch-clamp recordings from individual stereocilia of the auditory hair cells (55). Here, we demonstrate how angular approach HPICM can be used to image and guide the patch pipette to various regions of stereocilia at high resolution. Fig. 5 *A* shows a DIC image of the organ of Corti acquired using a 60× water immersion objective showing a row of outer hair cells (OHCs). The inset shows 2D projection of the topographical image of fixed OHCs (representative of 10, *n* = 10) acquired using the same angular approach HPICM setup with the scan head angle adjusted vertically (also shown in 3D view in Fig. 5 *B* together with the schematic representation of the pipette). An image of a live inner hair cell (IHC) acquired using the angular approach HPICM setup designed for integration into a patch-clamp setup on an inverted optical microscope is shown for comparison in Fig. 5 *C*.

When the HPICM pipette approaches the sample vertically, it has no side sensitivity (12, 13). Therefore, it cannot resolve vertical surfaces and surfaces located under overhanging structures. To demonstrate how angular approach HPICM can be used to visualize the vertical surfaces of stereocilia, we obtained images of OHC bundles with the pipette angled at 33° to the surface and the sample placed under a water-immersion objective of an upright microscope. Montages of an HPICM pipette schematic representation and real topographical data in coordinate system aligned with *XYZ* piezo axes are shown in Fig. 5, *D* and *F*, respectively. The corresponding 2D projections show stereocilia bundles from the inhibitory and excitatory directions (Figs. 5, *E* and *G*). To assess how the approach angle affects the vertical resolution of the angular approach HPICM, we imaged calibration samples with scan the head aligned both vertically and at a 45° angle. From the image of a calibration grid (Fig. 5 *H*) acquired using the angular approach HPICM with the scan head aligned vertically, we estimated the vertical noise of imaging to be around 7.6 nm measured as root mean-square. Cross-section profile (corresponding to the *white line* in Fig. 5 *H*) is shown in Fig. 5 *J*. The vertical noise calculated from the image of a similar calibration sample scanned at 45° angle to surface (not shown) was 19.2 nm root mean-square. Previously, we have shown that the XY resolution of HPICM depends on the nanopipette tip and could be at least as good as 20 nm for a standard, vertical approach setup (13). To demonstrate the lateral resolution of the angular approach HPICM that can be achieved in practice, we show 1 × 1 *μ*m scan clearly resolving individual 200 nm-wide stereocilia, measured as full width at half-maximum (FWHM), acquired with the pipette angled at 33° to the surface (Fig. 5 *I*). Cross-section profile (corresponding to the *white line* in Fig. 5 *I*) also shows 80 nm gaps between individual stereocilia measured at FWHM (Fig. 5 *K*, *hollow arrows*).

### Primary cultures of hippocampal neurons

**Figure 6:**
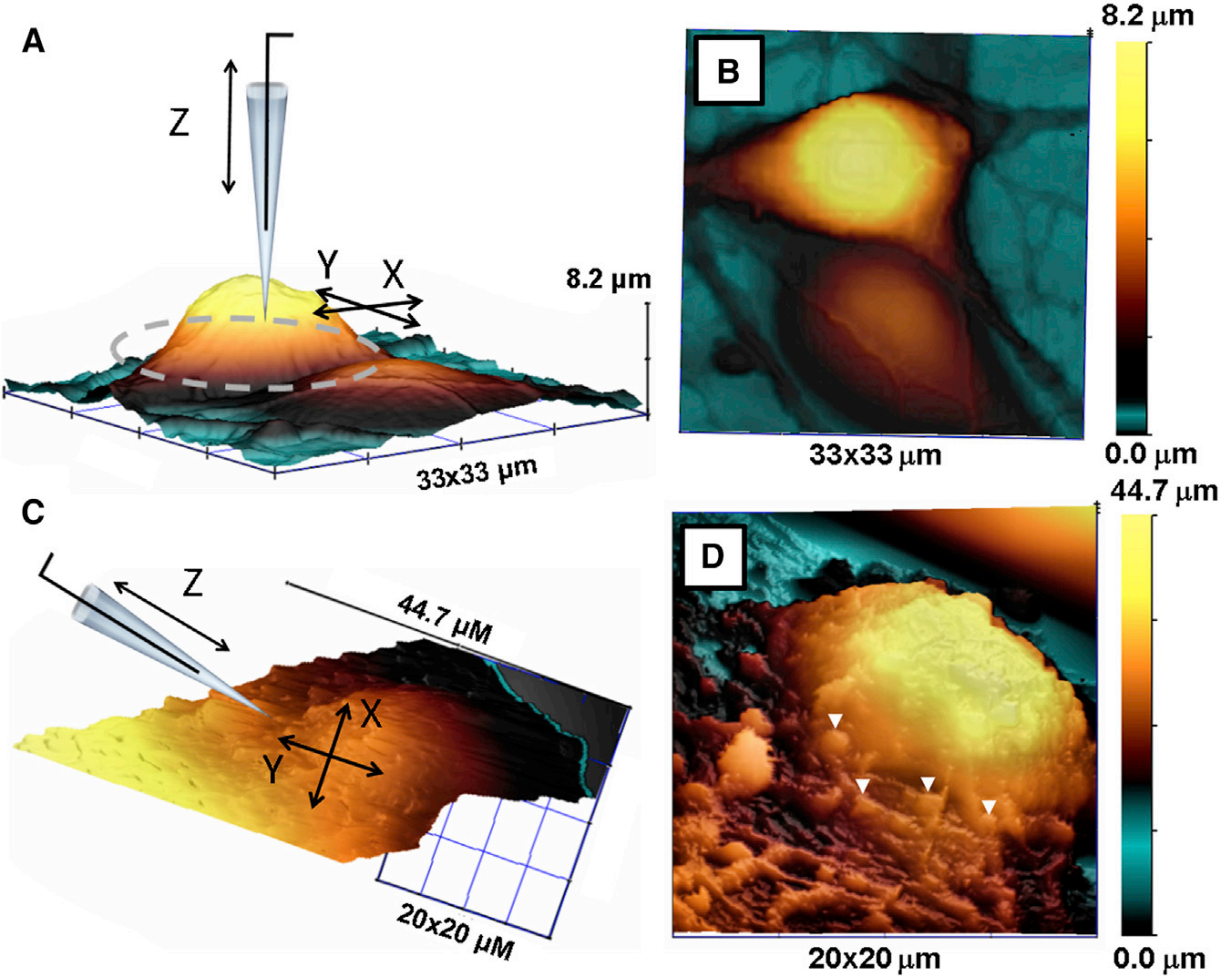
Imaging live primary hippocampal neurons of rat using HPICM on an upright optical microscope. (*A*) Montage of the topographical image of a hippocampal neuron and the schematic representation of a vertically aligned HPICM pipette. Dashed oval indicates steep slopes of soma unresolved by vertical HPICM. (*B*) 2D projection of the topographical image shown in (*A*). (*C*) Montage of the topographical image of a hippocampal neuron and the schematic representation of an HPICM pipette at an angle. Coordinate axes aligned with the axis of the pipette. (*D*) 2D projection of the topographical image shown in (*C*). To see this figure in color, go online.

We have previously demonstrated that the combination of HPICM and FCM could unambiguously identify active boutons and perform single-channel and whole-bouton recordings in primary hippocampal neurons (9). To further extend this approach and make it applicable to more physiologically relevant models, such as brain slices, we tested whether the HPICM with the adjustable approach angle could be used to image primary neurons under an upright optical microscope. Fig. 6 *A* shows a montage of the topographical image of a live hippocampal neuron and the schematic representation of a vertically aligned HPICM pipette. As in the previous examples, the vertically mounted HPICM pipette cannot resolve steep slopes on the edges of the neuronal soma (*dashed oval*).

Fig. 6 *B* shows 2D projection of the topographical image shown in Fig. 6 *A*. When the approach angle is set to 33°–45° to surface, HPICM topographical images (*n* = 3) cannot only provide side views, but also reveal structures resembling synaptic boutons (Fig. 6, *C* and *D*, *arrows*). More definitive identification of active synaptic boutons in brain slices will require combining HPICM with laser FCM.

### Cardiomyocytes

Another example of an area inaccessible to standard scanning probe imaging techniques is the intercalated disk (ICD) of cardiomyocyte. ICDs are highly organized components of cardiac muscle, which connect the ends of rod-shaped cardiac muscle cells and play a key role in maintaining mechanical and electric coupling between cardiomyocytes. The ICD is categorized into three major junctional complexes: desmosomes, fascia adherens junctions, and gap junctions (56). Although the importance of ICD gap junctions for cardiac function is not disputed, more recent work has shown that the intercalated disc also hosts a number of ion channels, including Na^+^ channels and K^+^ channels. Together with the proteins forming fascia adherens junctions and desmosomes (which interconnect actin filaments and intermediate filaments of adjacent cells), gap junction channels and ion channels form macromolecular protein complexes in the intercalated disc, each essential for cardiac function (57, 58). Recent experimental studies suggest regulatory interactions between these functional complexes and their involvement in the pathogenesis of important cardiac diseases (59). It had been demonstrated that Adenosine-triphosphate-sensitive K^+^ (K_ATP_) channel expression is concentrated at the ICD regions of ventricular myocytes, where they colocalize with desmosomal proteins (e.g., PG and PKP2). Immunohistochemistry experiments reveal that K_ATP_ channels also colocalize with other ICD proteins, such as N-cadherin, AnkG, and DP and to a lesser extent with the gap junction protein Cx43 (60). K_ATP_ channels are responsible for changes in the ATP/ADP ratio, providing a means to couple movement of potassium ions, and relate membrane potential with cellular energy status (61). Furthermore, they play an important part of endogenous protective signaling that promotes cellular survival under conditions of metabolic stress (62). A relationship between K_ATP_ channel activity and ischemia-induced conduction slowing has previously been noted experimentally (63). All these pieces of evidence suggest that desmosomal disorders might be associated with a diminished protective role of K_ATP_ channels.

**Figure 7:**
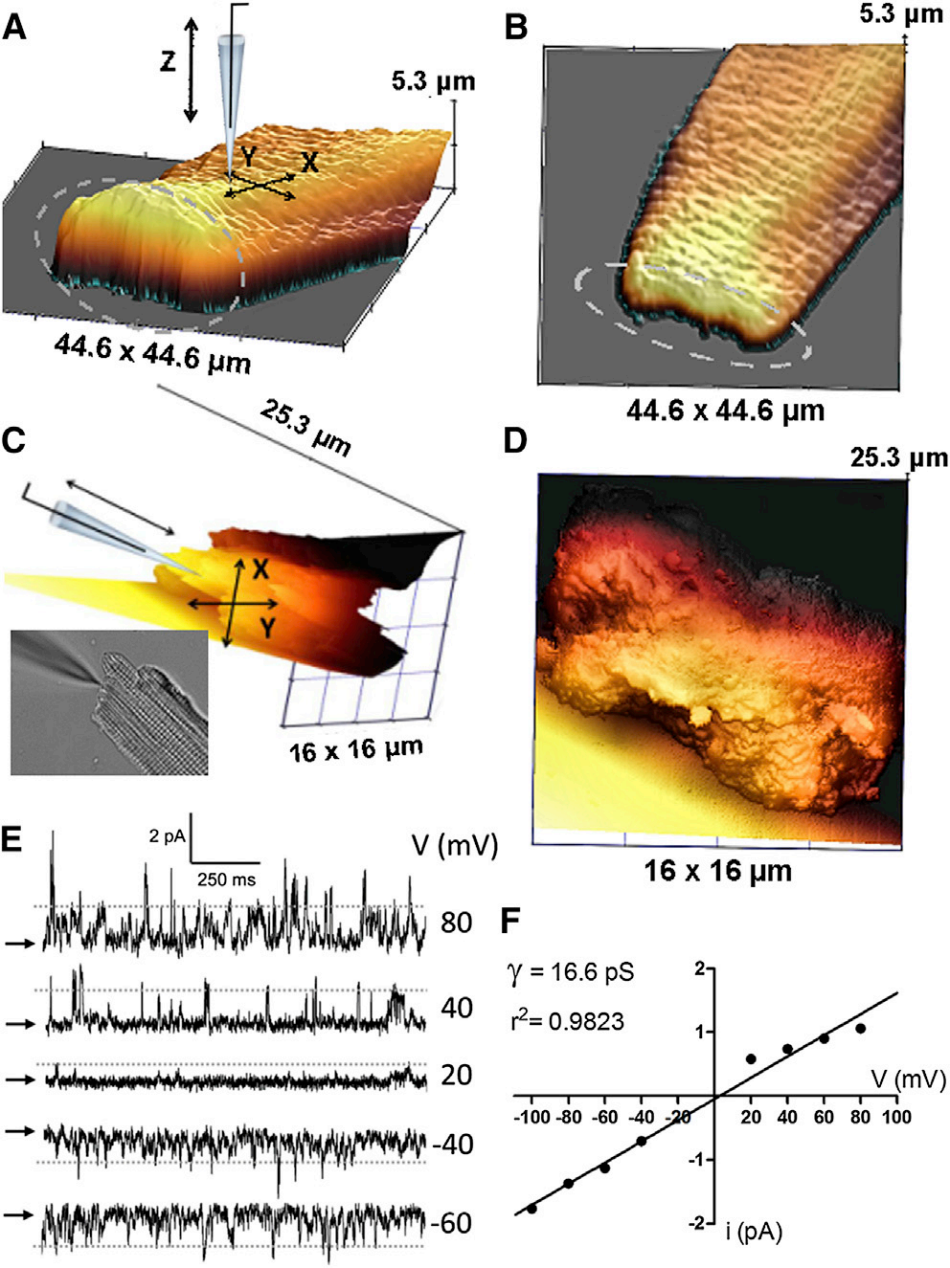
HPICM imaging and single-channel recordings at intercalated disks of isolated rat ventricular cardiomyocytes. (*A*) Montage of the topographical image of an adult rat cardiomyocyte and the schematic representation of a vertically aligned HPICM pipette. (*B*) 2D projection of the topographical image shown in (*A*). Dashed ovals indicate the intercalated disc unresolved by vertical HPICM. (*C*) Montage of the topographical image of an adult rat cardiomyocyte intercalated disk and the schematic representation of an HPICM pipette at an angle. Coordinate axes aligned with the axis of the pipette. Inset shows optical image of the cardiac myocyte and HPICM scanning/patching pipette acquired using 40× water immersion objective under an upright microscope. (*D*) 2D projection of the topographical image shown in (*C*). (*E*) Single-channel currents recorded from a cell-attached patch at various membrane potentials. Gray dotted lines represent the first level of conductance that was used to analyze the voltage dependence. (*F*) Voltage dependence of single K_ATP_ channels current. Linear fit corresponds to a single-channel conductance of 16.6 pS. To see this figure in color, go online.

Previously, ICDs of isolated cardiomyocytes could not be accessed using HPICM with vertical approach (Fig. 7, *A* and *B*, *dashed oval*).

Using HPICM with an adjustable approach angle, it is possible to produce images of intercalated disk surfaces. A representative image of 11 (*n* = 11) is shown in Fig. 7, *C* and *D*. We were also able to navigate the pipette and acquire single-channel recordings of K_ATP_ channels from the intercalated disk (Fig. 7 *E*). Fig. 6 *F* shows a current-voltage curve typical for K_ATP_ channels. Recent studies have shown a degree of heterogeneity of K_ATP_ channel distribution within a cardiac myocyte with the higher channel density at the intercalated disk that implies a possible role at the intercellular junctions during cardiac ischemia (60).

## Discussion

HPICM with angular approach now enables one to 1) seamlessly integrate HPICM into existing patch-clamp setups either on inverted or upright optical microscopes; 2) image cells grown on nontransparent substrates; 3) use DIC and phase contrast to image the cells comprising small organs and tissues; 4) image surfaces of cells previously inaccessible to scanning probes; 5) measure the angle at which the cell edge contacts the substrate, hence providing a better understanding of cell adhesion and spreading over the NNA; 6) use 3D topographical data for probe positioning to enable active interrogation of cells (e.g. electrophysiological recordings, localized application of drugs, etc.) with nanometer precision. This is a major technological advance for HPICM—the only imaging technique that can produce topographical images of living cells of such a great morphological diversity and complexity with nanometers resolution. Atomic force microscopy (AFM), another member of the scanning probe microscopy group—techniques that are used to directly measure geometrical characteristics of an object, is also frequently applied for topographical imaging of live cells. Although AFM is a benchmark technique capable of atomic resolution in the fields of physics and material sciences, the resolution it can achieve in living biological cells of mammalian origin under physiological conditions is still a matter of debate. The direct comparison of AFM and SICM for live cell imaging has demonstrated that, unlike in SICM where cell deformations are avoided because of the contact-free imaging mechanism, the apparent shape of whole cells in AFM depends on the imaging force, which deforms the cell (64, 65). As a result, the height of soft subcellular structures such as microvilli measured by AFM could be nearly three times lower than actual. Although imaging of living cochlear hair bundles of IHC and OHC has been demonstrated using custom-made AFM setup integrated with a DIC microscope, due to the nature of cantilever tip-sample interaction, the entire stereocilia could not be imaged, and only tips of stereocilia have been resolved (66, 67). It is also unclear how authors could claim better than 1 nm resolution given the fact that they used V-shaped cantilevers with typical radius of curvature 50 nm and tip angle 70°, and on average performed 200 scan lines per 4 × 4 *μ*m scan. It has recently been demonstrated that the imaging of the formation of clathrin-coated pits in living cells is possible by high-speed AFM using 5–8 nm sharp and 3 *μ*m long cantilevers (68). The observed pit diameter was 100 nm, which is similar to previously published work using HPICM (47) and is also in agreement with ample EM observations. To our knowledge, these are the highest resolution AFM images of living cells published to date. The extensive characterization of the nature of SICM image generation and resolution (69, 70) have shown that its topographical resolution depends on the angle between the pipette tip and the sample surface, and is highest when the pipette is normal to the surface (41). Therefore, the possibility of adjusting the HPICM pipette approach angle enables the most optimum setting for the high resolution imaging of steep slopes and vertical surfaces of living cells. Here, we demonstrate this advantage by showing the images of individual 200 nm diameter stereocilia and clearly resolved 80 nm gaps between individual stereocilia.

By imaging a diverse spectrum of samples, we demonstrate and prove that HPICM with angle approach can contribute to many fields, such as nanomedicine, endocrinology, cell physiology, and neuroscience. Recently published research has already demonstrated how angle approach HPICM, in cohort with other powerful imaging techniques, could be used to study the clusterization of the adhesion molecule N-cadherin and the voltage-gated sodium channels in intercalated discs of cardiac myocytes (71). We aim to extend the functionality of the angular approach HPICM by combining it with a synchronized fluorescence excitation-detection system that will enable us to apply the methodology of nanoscale-targeted patch-clamp recordings originally developed and validated on primary hippocampal neuron cultures (9) to brain slices, as a more physiologically relevant model. Recently, we have developed a range of nanopipette-based sensors that could be formed at the tip of one barrel of a double-barrel nanopipette, leaving another barrel free for independent simultaneous ion conductance measurement required for probe-sample distance regulation (72, 73, 74). These probes could be used for multiparametric HPICM imaging to deliver such important data as, for example, localized oxygen consumption or nitric oxide concentration and correlate them to live high-resolution topography. Angular approach HPICM nanopipette could also provide a rapid quantitative molecule delivery using previously described protocols based on hydrostatic pressure and/or voltage application (74, 75). Such a tool could be used to locally expose cells to defined concentrations of substances enabling drug discovery/testing experiments. These advances will result in a versatile and flexible scanning probe imaging system that could be easily integrated into standard electrophysiological setups used in many laboratories around the world and find a wide range of applications in live imaging.

## Author Contributions

A.S. designed research, performed research, analyzed data, and wrote the article. S.T. performed research, analyzed data, and wrote the article. S.G., J.L.-S.-A., and A.C.V.-O. performed the research. A.I.T. performed the research and wrote the article. P.R., C.C., M.M.S., J.G., and G.I.F. wrote the article. D.K. designed research and wrote the article. Y.E.K. designed research, contributed analytic tools, and wrote the article.
